# Supplementary material for: Endometrial congestion is the only hysteroscopic finding indicative of chronic endometritis
Source: PLoS One. 2024 Jun 27;19(6):e0303041. doi: 10.1371/journal.pone.0303041 (PMC11210814; doi:10.1371/journal.pone.0303041)
Supplement: Attachment — Submitted filename: Response to Reviewers.docx [file pone.0303041.s002.docx]

March 22, 2024

Muhammad Salman Bashir, M.S.C Editor-in-Chief

Academic Editor

*PLOS ONE*

Dear Editor:

We are pleased to submit the revised version of our manuscript entitled, “Endometrial congestion is the only true hysteroscopic finding suggestive of chronic endometritis.” The submission ID is PONE-D-24-02159.

We thank you and the reviewers for your helpful comments and feedback. The manuscript has benefited from these insightful suggestions. The manuscript has been carefully re-checked and we have made the necessary changes according to your comments. We look forward to working with you and the reviewers to move this manuscript closer to publication in *PLOS ONE.*

The revisions in the manuscript are highlighted in yellow to facilitate the review process. Please also find below our point-by-point responses to all your comments. We hope that our explanations and corrections are satisfactory.

We look forward to hearing from you at your earliest convenience.

Sincerely,

Ayumu Ito, MD, PhD

Department of Obstetrics and Gynecology, Faculty of Medicine, Toho University

6-11-1, Omorinishi, Ota-ku, Tokyo, Japan

Phone: +813-3762-4151

Email: [ayumu.itou@med.toho-u.ac.jp](mailto:ayumu.itou@med.toho-u.ac.jp)

**Responses to the editorial team**

We are grateful for the critical comments and insightful suggestions, which have helped us improve our manuscript. We have integrated all your comments and suggestions in the revised version of our manuscript.

**[Comment]**

1. Please ensure that your manuscript meets PLOS ONE's style requirements, including those for file naming. The PLOS ONE style templates can be found at

https://journals.plos.org/plosone/s/file?id=wjVg/PLOSOne_formatting_sample_main_body.pdf and

https://journals.plos.org/plosone/s/file?id=ba62/PLOSOne_formatting_sample_title_authors_affiliations.pdf

**[Response]**

We have verified that this manuscript complies with the style guidelines of PLOS ONE.

**[Comment]**

2. In this instance it seems there may be acceptable restrictions in place that prevent the public sharing of your minimal data. However, in line with our goal of ensuring long-term data availability to all interested researchers, PLOS’ Data Policy states that authors cannot be the sole named individuals responsible for ensuring data access (http://journals.plos.org/plosone/s/data-availability#loc-acceptable-data-sharing-methods).

Data requests to a non-author institutional point of contact, such as a data access or ethics committee, helps guarantee long term stability and availability of data. Providing interested researchers with a durable point of contact ensures data will be accessible even if an author changes email addresses, institutions, or becomes unavailable to answer requests.

Before we proceed with your manuscript, please also provide non-author contact information (phone/email/hyperlink) for a data access committee, ethics committee, or other institutional body to which data requests may be sent. If no institutional body is available to respond to requests for your minimal data, please consider if there any institutional representatives who did not collaborate in the study, and are not listed as authors on the manuscript, who would be able to hold the data and respond to external requests for data access? If so, please provide their contact information (i.e., email address). Please also provide details on how you will ensure persistent or long-term data storage and availability.

**[Response]**

We have provided all data generated or analyzed in this study as supporting information files.

**[Comment]**

3. Please review your reference list to ensure that it is complete and correct. If you have cited papers that have been retracted, please include the rationale for doing so in the manuscript text, or remove these references and replace them with relevant current references. Any changes to the reference list should be mentioned in the rebuttal letter that accompanies your revised manuscript. If you need to cite a retracted article, indicate the article’s retracted status in the References list and also include a citation and full reference for the retraction notice.

**[Response]**

We have verified that the reference list is complete and correct and that it meets the submission guidelines.

**Responses to the Reviewers**

We would like to thank you for your critical comments and insightful suggestions, which have helped us improve our manuscript quality. As indicated in the responses below, we have integrated all your comments and suggestions in the revised version of our manuscript.

**Reviewer 1**

**[Comment]**

1- in demographic information many items wre missing ( number IVF trial , history of abortion, received antibiotics, ..etc).

**[Response]**

Thank you raising this issue. We have added the information regarding the history of parity and abortion based on our data to table1. Unfortunately, we do not have data on the number of IVF trials or whether antibiotics were administered; therefore, we could not include this information. We would be grateful for any further advice or suggestions you might have.

**[Comment]**

2- the abstract need to be summarized.

**[Response]**

We have condensed the abstract as suggested.

**Reviewer 2**

**[Comment]**

1. - firstly, I advise you to take into account this paper to improve you reference quality with the most recent papers published on this topic (doi: 10.1007/s00404-023-07163-w;

- What could it be the solution about adhesions and their possible treatment? (doi: 10.1016/j.ajog.2021.09.015)

**[Response]**

Thank you for your suggestions. Following your recommendation, we have added one of the suggested papers to line 207, page 10 of the revised manuscript.

In this study, we have not investigated adhesions; therefore, we chose to omit any mention of them. We hope for your understanding on this matter.

**[Comment]**

2. Line 79: the uterine cavity

**[Response]**

We have corrected the text accordingly (line 95, page 5 of the revised manuscript).

**[Comment]**

3. Line 88: In our Institution

**[Response]**

We have corrected the text accordingly (line 85, page 4 of the revised manuscript).

**[Comment]**

4. In general, english should be revised by mother tongue

**[Response]**

Thank you for your feedback. The revised manuscript has been reviewed by a native English speaker.

**[Comment]**

5. Regarding "Hysteroscopic findings of the uterine lumen" (edit with cavity), what do you think it is more important?

**[Response]**

We consider that endometrial congestion is the most significant hysteroscopic finding in the uterine lumen indicative of chronic endometritis. To clarify this point, we have added a conclusion at the end of the Discussion section.
